# Prognostic value of increase in transcript levels of Tp73 ΔEx2-3 isoforms in low-grade glioma patients

**DOI:** 10.1038/sj.bjc.6603410

**Published:** 2006-10-17

**Authors:** M Wager, J Guilhot, J-L Blanc, S Ferrand, S Milin, B Bataille, F Lapierre, S Denis, T Chantereau, C-J Larsen, L Karayan-Tapon

**Affiliations:** 1 Neurosurgery Department, University Hospital, Poitiers, France; 2 Clinical Research Centre, University Hospital, Poitiers, France; 3 Department of Pathology, University Hospital, Poitiers, France; 4 Laboratory of Molecular Oncology, University Hospital, Poitiers, France

**Keywords:** Tp73 isoforms, low-grade gliomas, prognosis, quantitative reverse-transcription polymerase chain reaction (QRT-PCR)

## Abstract

Glial tumours are a devastating, poorly understood condition carrying a gloomy prognosis for which clinicians sorely lack reliable predictive parameters facilitating a sound treatment strategy. Tp73, a p53 family member, expresses two main classes of isoforms – transactivatory activity (TA)p73 and ΔTAp73 – exhibiting tumour suppressor gene and oncogene properties, respectively. The authors examined their expression status in high- and low-grade adult gliomas. Isoform-specific real-time reverse transcription-polymerase chain reaction was used for the analysis of Tp73 isoform transcript expression in a series of 51 adult patients harbouring glial tumours, in order to compare tumour grades with each other, and with non-tumoural samples obtained from epileptic patients as well. Our data demonstrate increase of TAp73 and ΔTAp73 transcript levels at onset and early stage of the disease. We also show that ΔEx2–3 isoform expression in low-grade tumours anticipates clinical and imaging progression to higher grades, and correlates to the patients’ survival. Expression levels of P1 promoter generated Tp73 isoforms – and particularly ΔEx2–3 – indeed allow for prediction of the clinical progression of low-grade gliomas in adults. Our data are the first such molecular biology report regarding low-grade tumours and as such should be of help for sound decision-making.

Glioblastomas are divided into primary or *de novo*, and secondary tumours originating from low-grade (World Health Organization – WHO – grade II) progressing to high-grades, that is anaplastic (WHO grade III) gliomas and eventually to highly malignant glioblastomas (WHO grade IV). Although our knowledge of the molecular mechanisms involved in tumorigenesis and progression has significantly improved over fairly recent years, this has yet to result in an improved therapeutic strategy. To date, no reliable parameter has allowed for anticipation of clinical progression from lower to higher grades. Such criteria would be of paramount importance in therapeutic management of these patients.

The Tp73 gene, a close relative of the tumour suppressor Tp53 gene, contains two independent promoters P1 and P2 (Melino *et al*, 2002). P1 promoter controls the TAp73 transcripts containing exons 1–3 that encode N-terminal sequences with transactivatory activity (TA), and ΔEx2, ΔEx2–3 and ΔN’ transcripts collectively designate ΔTAp73 that lack a fully competent TA domain (Stiewe *et al*, 2002a, 2002b). The TAp73 isoform is capable of inducing cell cycle growth arrest and apoptosis and is also involved in the response of p53 to death stimuli (Flores *et al*, 2002). These features are consistent with a tumour suppressor function. In contrast, the ΔTA transcripts do not induce cell growth arrest or cell death. Significantly, their overexpression in NIH3T3 cells induces malignant transformation and tumour growth in nude mice (Stiewe *et al*, 2002a, 2002b), which is consistent with an oncogene status. The P2 promoter located downstream of exon 3′ controls ΔNp73 transcripts lacking the TA domain that likewise exhibit antiapoptotic activity and are therefore functionally similar to ΔTAp73 transcripts (Grob *et al*, 2001; Nakagawa *et al*, 2002). ΔNp73 has been shown to facilitate the immortalisation of MEFs and to cooperate with oncogenic Ras in transforming primary fibroblasts *in vitro* and in inducing MEF-derived fibrosarcomas *in vivo* in nude mice (Petrenko *et al*, 2003). Because the tetramerisation domain and the DNA-binding domain of the TAp73 protein are conserved in ΔNp73 and ΔTAp73 isoforms, both have been shown to exert a dominant-negative activity on TAp73 and p53 through oligomerisation with the two proteins and/or by competition for p53/TAp73 target genes (Stiewe *et al*, 2002a, 2002b). Consistent with these data, upregulation of the ΔNp73 isoform has been described in neuroblastomas with poor prognosis and thereby defines this parameter as a strong and independent predictor of such poor prognosis (Casciano *et al*, 2002). Moreover Bergamaschi *et al* (2003) and Irwin *et al* (2003) have shown that TAp73 is induced by a wide variety of chemotherapeutic agents and that chemosensitivity is related to TAp73 function.

In view of the potential relevance of Tp73 to tumorigenesis, the expression status of the gene has been addressed in various tumour types. Significant results have been achieved only in more recent works taking into account the recently described ΔTAp73 transcripts by using appropriate couples of primers, designed to specifically assess contribution of the ΔTAp73 isoforms among the different P1-generated transcripts collectively referred to as P1 transcripts (Ng *et al*, 2000; Cui *et al*, 2005). The most convincing evidence to date comes from a work on a series of 100 ovarian tumours of all the known histological types (Concin *et al*, 2004). On the whole, these data point to the activation of the P1 promoter and the deregulation of P1-controlled ΔTA p73 isoforms in the tumorigenic process. Furthermore, because the ΔN’p73 isoform is upregulated in ovarian tumours but not in other cancers, the data argue in favour of a selective deregulation among the P1 transcripts in accordance with types of cancer.

We have undertaken a prospective study in order to determine the involvement of Tp73 isoforms in gliomagenesis and their prognostic relevance. In the first part we performed a global analysis of P1- and P2- generated transcripts on high- and low-grade gliomas, whereas in the second part we focused on low-grade gliomas and performed a discriminant analysis of P1- generated transcripts.

## PATIENTS AND METHODS

### Patients

Tissues from 51 adult patients harbouring glial tumours were successively collected during surgery at the Department of Neurological Surgery, University of Poitiers, France, with signed informed consent of all patients and the approval of the ethics committee of the Poitou-Charentes area. These patients were free from any past medical history, especially with regards to brain surgery, brain radiation therapy or chemotherapy. Our series also included three tumour-free patients, operated for refractory epilepsy, obtained from Neurobiotec® (Lyon, France) in compliance with the ethics committee. For each patient a sample was collected at the presumed higher-grade region and named the ‘central’ sample. Whenever deemed reasonable – that is without significant additional functional risk – a second sample was harvested in the vicinity of the tumour and called ‘peripheral’.

All patients were treated between January 2002 and May 2003. Distribution of patients in groups according to the WHO pathology classification appears in Table 1. Twenty-nine patients underwent tumour resection, whereas 22 benefited from stereotactic biopsy. The first series, dedicated to Tp73 involvement, included 28 high-grade and 11 low-grade patients (Table 1A), and 12 were added to this group in a second series focusing on Tp73 isoforms (Table 1B). All low-grade patients had non-enhancing tumours on MRI imaging, and pathology-proven low-grade tumours. In all cases progression to a higher grade was pathology-proven. Tumour diagnosis and grading were established according to the WHO criteria (Kleihues and Cavenee, 2000) and were systematically revised by two expert neuropathologists.

### Analysis of Tp73 isoform transcripts

#### Tissue harvesting and preparation

Per operative pathology exam allowed to check glial tumour diagnosis with *s*amples obtained either from open-sky surgery or stereotactic biopsies. Each tumour sample was divided into two parts: one was dedicated to smears, the second was immediately frozen in liquid nitrogen in the operating room, and stored at –80°C until usage.

#### RNA isolation and complementary DNA preparation

Total RNA was extracted from tumour tissues using the RNAeasy® Mini Kit (Qiagen, Courtaboeuf, Paris, France) according to the manufacturer's instructions with minor modifications, for exclusion of contaminated genomic DNA. The spin-column membranes were treated with DNase (Qiagen) for 15 min at room temperature before elution. Ten microliters of DNAse-treated total RNA was transcribed into cDNA using Superscript™ II RnaseH - and random hexamers (Invitrogene®, Courtaboeuf, Paris, France).

#### Real-time reverse transcription-polymerase chain reaction and relative quantification

**Figure 1:**
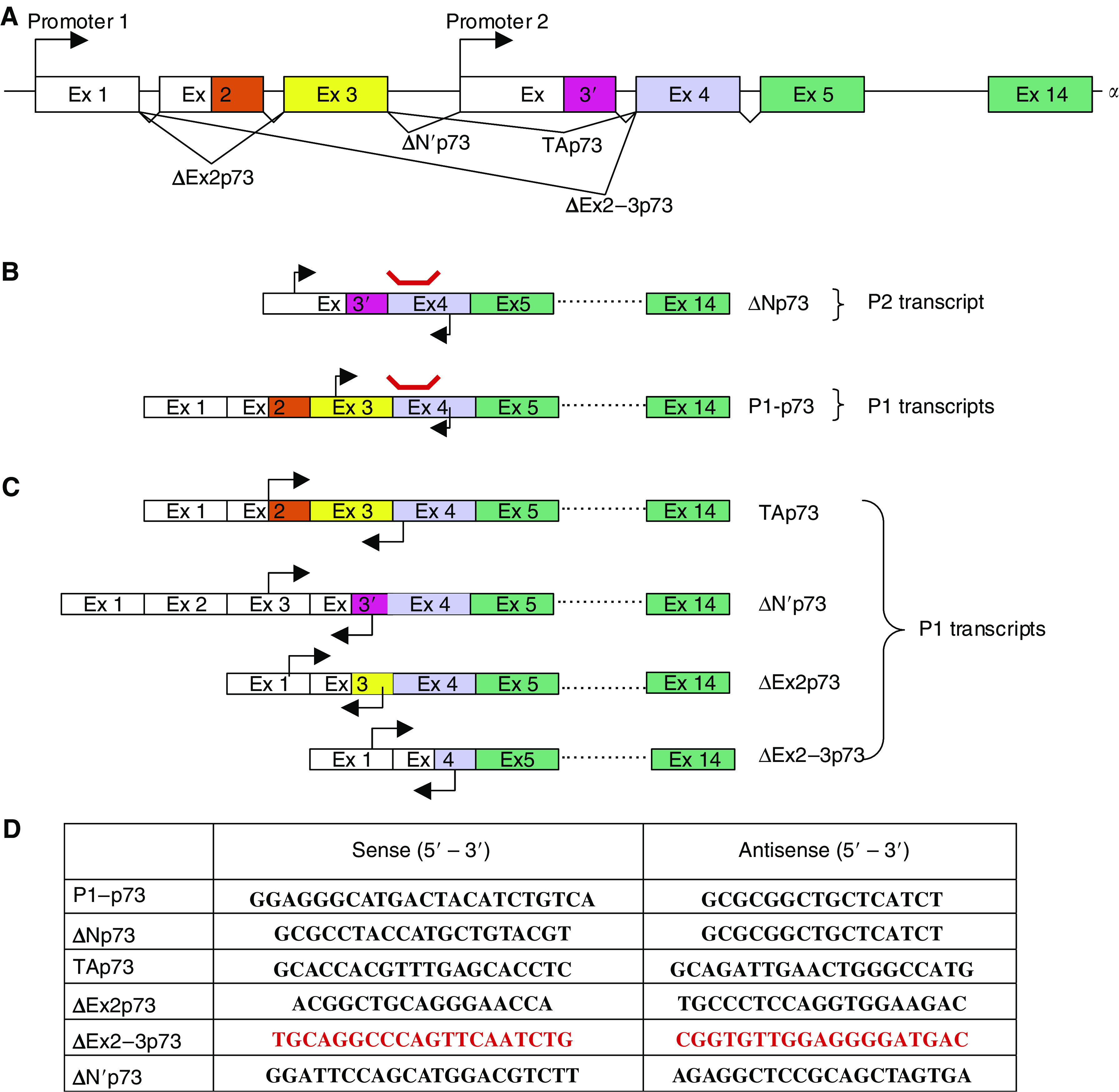
(**A**) Genomic organisation of the NH2 terminus of Tp73. Arrows indicate transcriptional start sites. Exons are represented as boxes. The colours indicate the number of the exons, and white untranslated sequences. Exons 2 and 3 encode for the transactivation domain of the full-length p73 protein. Spliced transcripts regulated by Promoter 1 are labelled TAp3, ΔEx2p73, ΔEx2–3p73 and ΔN’p73. (**B**) Localisation of the primer pairs (arrows) and probe (red line) used in the global analysis of promoter 1 encoded transcripts (P1–p73) and promoter 2 encoded transcript (ΔNp73) by real-time PCR assays. (**C**) Localisation of the primer pairs (arrows) used for isoform-specific real-time PCR assays. (**D**) Sequences of the sense- and antisense-primers used for the global analysis of promoter 1 generated transcripts (P1–p73) and promoter 2 generated transcript (ΔNp73) and those of isoform-specific amplification of individual NH2 terminus Tp73 isoforms. The forward primers for ΔEx2p73 as well as for ΔEx2–3p73 were designed on the exon-exon boundary (exon1/exon3 for ΔEx2p73 and exon1/exon4 for ΔEx2–3p73). These primers could not hybridise on TAp73 transcripts as the boundary exists only in the variants forms. The forward primer used for specific TAp73 amplification is localised in exon 2 and the reverse on the exon3/exon4 boundary and could only amplifiy TAp73. Specificity for ΔN’p73 was achieved by the unique combination of the upstream (on exon3) and downstream (on exon 3′) primers.

We assessed levels of Tp73 isoform mRNA transcripts by real-time quantitative PCR in the ABI PRISM 7000 sequence detection system (Applera, Courtaboeuf, Paris, France). Primer sequences are listed in Figure 1. All primers were queried against the no redundant Human Genome Database. Probe sequence is 5′-CAGTTCAATCTGCTGAGCA-3′. All primer pairs detected a unique specific cDNA. The forward primers for ΔEx2p73 as well as for ΔEx2–3p73 were designed to specifically recognise on the exon–exon boundaries (exon1/exon3 for ΔEx2p73 and exon1/exon4 for ΔEx2–3p73). Indeed, these primers could not hybridise on TAp73 transcripts as the selected boundaries exist only in the variants forms. The forward primer used for specific TAp73 amplification is localised in exon 2 and the reverse on the exon3/exon4 boundary and could only amplify TAp73. Specificity for ΔN’p73 was achieved by the unique combination of the upstream (on exon3) and downstream (on exon 3′) primers. However, the specific amplification of TAp73, ΔEx2p73, ΔEx2–3p73 and ΔN’p73 transcripts was checked on gel and gives as expected a single band of 168, 115, 178 and 90 pb. This specific amplification was confirmed by sequencing the products.

The reactions were carried out first by using the Taqman® chemistry for the global screening of P1-generated p73 transcripts (P1–p73) and P2-generated transcripts (ΔNp73). We then used SyberGreen chemistry assessing of discriminant Tp73 isoform expression. Briefly, the PCR reactions were performed in 25 *μ*l reaction volumes consisting of 1 × Taqman Universal PCR Master-Mix or SyberGreen Universal PCR Master-Mix (Applera), one out of 20 of the reverse transcription reaction, 300 nm of each primer and 200 nm of probe when Taqman chemistry was used. The samples were submitted to amplification as follows: heating at 50°C for 2 min, 95°C for 10 min followed by 40 cycles at 95°C × 15 s, 60°C × 1 min. Each RNA sample was tested in duplicate and a negative control was included in every plate. The computed tomography value was defined as the cycle number (*C*_t_) at which the fluorescence crossed the threshold. Range of threshold cycles was *C*_t_: 25–37.6 for P1–TAp73, *C*_t_: 26.9–39.8 for ΔNp73 *C*_t_: 22.89–39.43 for TAp73, *C*_t_: 26.73–37.33 for ΔEx2p73, *C*_t_: 23.24–38.75 for ΔEx2–3p73 and *C*_t_:30.32–36.36 for ΔN’p73. Results were normalised using the endogenous reference GAPDH. The threshold cycle differences is given in Table 1. The amplification efficiency of the reference gene was similar to that of the target genes. We employed the relative quantification method described in Applied Biosystems User Bulletin No. 2 (Applied Biosystem User Bulletin, 1997) and by Livak and Schmittgen (2001), in which the amount of target, normalised to the endogenous reference GAPDH and relative to the non-tumoural epileptic tissue, is indicated by the 2^−ΔΔ*C*t^ formula where ΔΔ*C*_t_=Δ*C*_tTumoural_−Δ*C*_tNonTumoural_.

### Statistical analysis

The data distribution of expression levels being non-Gaussian, non-parametric tests were used for data analyses. Increase in transcript levels of each Tp73 isoform was analysed by signed tests, whereas differences between tumour tissues were tested by the Wilcoxon rank sum test. Each isoform was dichotomised according to its median value. Survival was estimated according to the Kaplan–Meier method and compared between groups by means of the log-rank test. For correlations among the various isoforms and patient age, the threshold cycle differences were used and the Spearman correlation test was applied. Multivariate analyses were performed with the Cox proportional hazard model. Models were fitted for the threshold cycle differences and for the dichotomised variable. All tests were two-sided and the type I error was set at 5%. Statistical analyses were performed with use of SAS Statistical Software (Release 8.02).

## RESULTS

Global analysis of P1 – (P1–p73) and P2 – (ΔNp73) generated transcript expression levels conducted in 11 patients shows that P1–p73 transcript levels are increased in low-grade gliomas (*P*<10^−3^) when compared to epileptic tissue samples taken as non-tumoural controls (Table 1C). In contrast, tumour samples displayed no increase of ΔNp73 transcript levels. The same observation was carried out in 28 high-grade gliomas (*P*<10^−3^). Because no significant difference was found in P1–p73 transcript expression between high- and low-grade tumours, deregulation of the P1 promoter that generates the TAp73 and ΔTAp73 isoforms was likely to occur early in the tumorigenic process. This upregulation of the P1–p73 transcripts was observed in the centre of the tumours whatever their grade. In contrast, although the high-grade tumour periphery still displayed high levels of the transcript, this expression was significantly (*P*=0.031) lower on the periphery of low-grade tumours (Table 2).

On the whole, these data suggest that low- and high-grade tumours can be distinguished on the basis of their P1–p73 transcript expression status in their central and peripheral areas. In addition this selective expression of P1–p73 between these areas in low-grade tumours argues in favour of an evolving process.

**Figure 2:**
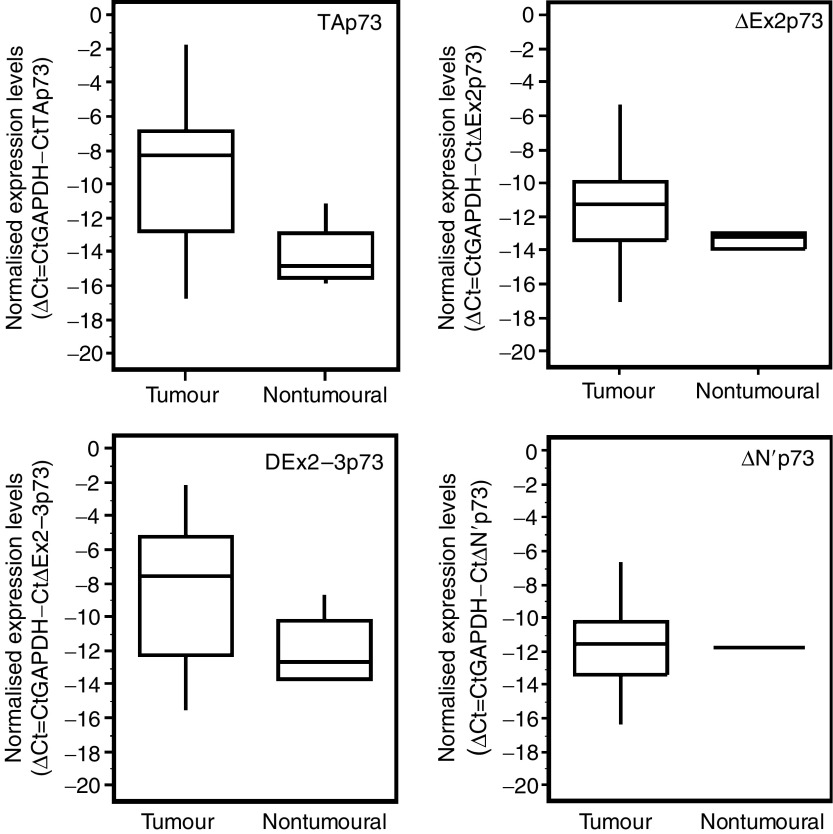
Box plot diagrams showing the normalised expression levels Δ*C*_t_ (CtGAPDH-gene) of promoter 1- generated Tp73 isoforms (TAp73, ΔEx2p73, ΔEx2–3p73 and ΔN’p73) in 23 low-grade gliomas tumours and three non-tumoural tissues. The line within the boxes indicates the median expression level. The top edge of the boxes represent the 75th percentile, the bottom edge, the 25th percentile. The range is shown as a vertical edge.

Analysis of P1-p73 transcripts (TAp73, ΔEx2p73 and ΔEx2–3p73 and ΔN’p73) shows that TAp73, ΔEx2p73 and ΔEx2-3p73 transcripts levels were increased (*P*<0.05) as compared to the expression spectrum displayed by non-tumoural, control tissue (Table 1C and Figure 2). On the contrary, expression of the ΔN’ isoform in tumour samples did not appear to differ from that of controls. In addition, expressions of these different isoforms were correlated to each other as significant associations were observed between TAp73 and ΔEx2p73 levels (*P*<10^−2^) and TAp73 and ΔEx2–3p73 levels (*P*<10^−3^). These data led to the conclusion that, with the exception of the ΔN’ isoform, all P1 transcripts were upregulated in the tumours. Again, these findings argued in favour of the early deregulation of the P1 promoter in tumours.

Differences in the expression levels were observed within tumoural and non-tumoural tissue samples. This observation was particularly noticeable for TAp73 and ΔEx2–3p73 transcript levels with a variation of >30 000-fold and >10 000-fold within this groups (lowest versus highest expression). A similar observation was reported by Concin *et al*, 2004 concerning ovarian carcinomas where ΔEx2–3p73 show in normal tissues a variation of >7000-fold from the lowest to highest measured. One possible explanation for this variation range is that this variation may reflects perceptibly the heterogeneity of the tumours, patient-to-patient variability or both.

**Figure 3:**
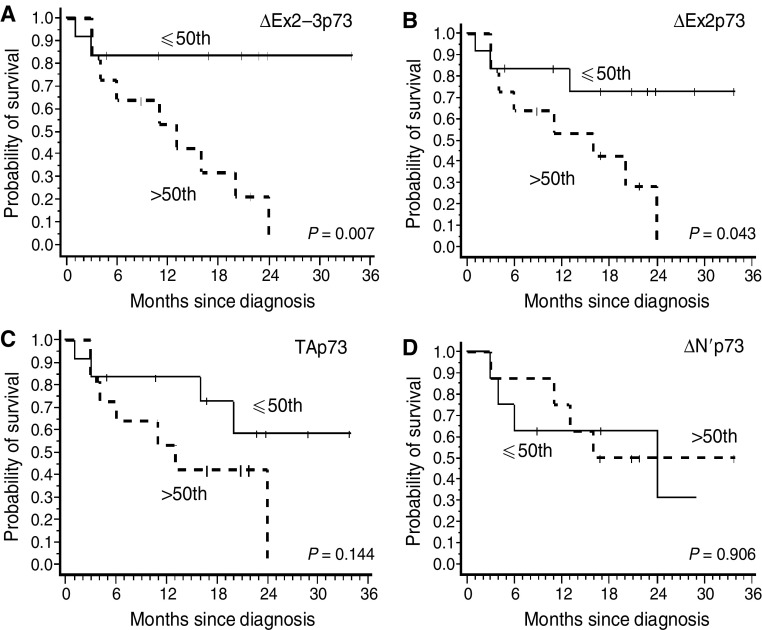
Kaplan–Meier survival curves in 23 low-grade gliomas. Two groups were defined taking as reference the median values of the mRNA levels of different transcripts (above and below the 50th percentile of the range of the different transcripts). (**A**) ΔEx 2–3p73, (**B**) ΔEx2p73, (**C**) TAp3 and (**D**) ΔN’p73.

In order to determine the clinical relevance of these findings with regards to low-grade glioma outcome, survival analysis was performed as regards overall survival rates. Taking as references the median values of mRNA levels of different transcripts – above and below the 50th percentile of the range of the latter – two groups were defined. This approach was dictated by the impossibility of estimating the absolute copy numbers of transcripts. Group 1 included patients in whom the expression levels for each transcript species were higher than their respective median values. Group 2 included patients whose expression levels were lower or equal to the median values. Among the isoforms, expression levels of the ΔEx2–3p73 transcript proved to be a strong prognostic marker for overall survival in our low-grade glioma cohort (*P*=0.007, Figure 3A). Although 10 out of 12 (83%) ΔEx2–3p73 low expressor patients (group 2) were still alive as of the last follow-up, nine out of eleven (82%) of the high expressor (group 1) patients were deceased. The median overall survival time for high expressor patients was 13 months (IC95% 4–20) whereas median survival time for low expressor patients was not reached. The high expresssor patients (group 1) had a relative amount of ΔEx2-3p73 mRNA >22-fold compared with non-tumoural epileptic tissue whereas patients from group 2 had a relative amount of ΔEx2–3p73 mRNA <=22-fold as compared to non-tumoural epileptic tissue.

In multivariate analysis including age and ΔEx2–3p7 expression, age is a significant prognostic factor (*P*=0.017) and a trend is observed for ΔEx2–3p73 (*P*=0.070). However, when ΔEx2–3 expression is classified in low and high groups, the only factor that remains significant, even when age is taken into account, is ΔEx2–3p73 (*P*=0.025).

A similar observation was also carried out, but to a lesser extent, for ΔEx2p73 as high expressor patients had a worse outcome than did low expressor patients (*P*=0.043 and Figure 3B). In contrast, TAp73 and ΔN’p73 transcripts did not appear to be involved as the survival rates in the two groups were similar (*P*=0.144 and *P*=0.906, respectively Figure 3C and D).

As we know that the neutralising role of these transdominant forms take place through oligomerisation with TAp73 and p53 proteins it is crucial to confirm that RNA overexpression of the pronostic splice variants as far as proteins are concerned. Unfortunately no specific antibodies for any of the ΔTAp73 forms currently exist. We cannot therefore propose the existence of a dominant negative mechanism with ΔEx2–3p73 and ΔEx2p73 variants in low-grade gliomas. However, as mRNA expression of ΔEx2–3p73 and to a lesser extent ΔEx2p73 appeared to be correlated to low-grade gliomas patients survival and to be a strong prognostic marker in these tumours, our data, although suggestive on the nefastious role of these transcripts, do not allow to definitely conclude on this point.

## DISCUSSION

### Tp73 involvement in glial tumorigenesis

The role of the Tp73 gene in oncogenic process has been underlined by several reports dealing with different tumour types. Results including ours have clearly shown that the expression of the gene rises frequently in a number of tumours although considerable variations in the levels of transcripts from patient to patient can be noted (Concin *et al*, 2004). A previous work dealing with gliomas and using a semiquantitative RT-PCR assay has shown that a similar situation also exists in this type of tumour as overexpression of TAp73 mRNA occurred in high-grade gliomas whereas only a few tumours displayed ΔNp73 expression (Ugur *et al*, 2004). The present study confirms these data and extends them by reporting increase in transcript levels of the P1-generated Tp73 transcripts, namely TAp73, ΔEx2p73 and ΔEx2-3p73, (but not ΔN’p73) in a cohort of low-grade adult gliomas. Interestingly ΔNp73 transcript does not appear to differ from that of control samples. Thus, deregulation of the P1 promoter and emergence of particular RNA populations are likely to be an early event as this deregulation is likewise observed in high-grade gliomas and could possibly influence cell evolution toward malignancy.

The abundance of Tp73 mRNA isoforms in specific cell type is complex and is likely to result from differential expression, RNA stability and splicing. In our study, increase in transcript levels of P1-generated isoforms could be explained by coupled mechanisms: activation of the P1 promoter itself and selective splicing process leading to an increase of certain mRNA species (TAp73, ΔEx2p73 and ΔEx2–3p73 but not ΔN’p73). The expression of some transcripts could be restricted to particular areas of the tumour suggesting differential splicing in this area. Addressing this question would require to perform RNA status analysis of at the individual tumour cells level.

The selective patterns of expression of the transdominantTp73 gene isoforms observed in a variety of tumours (Ng *et al*, 2000; Cui *et al*, 2005), should reflect a particular distribution of the factors that contribute to splicing such as HnRNP proteins. Incidentally, several reports have shown the existence of particular populations of HnRNP proteins in a variety of tumours suggesting that their expression is implicated in selective activation of gene isoforms expression (Carpenter *et al*, 2006).

Tumour-specific variants have been reported to affect transcript stability and thus, accumulation of the variant transcripts could result from an increased stability. Regarding this point, impaired mRNA turnover and stability are shown to play a critical role in the activation of specific genes during the cellular response to mitogens, immunological triggers stressful stimuli and differentiation agents (Bashirullah *et al*, 2001; Fan *et al*, 2002). Several parameters control the mRNA turnover: RNA-binding proteins (RBPs) that bind to specific RNA sequences and either increase or decrease the transcript half-life (Brennan and Steitz, 2001; Gorospe, 2003;); regions located in the 3′-untranslated regions (3′-UTRs) of mRNA. In the latter, differences in 3′-UTRs of splice variants could be recognised by proteins expressed specifically in some normal tissues or tumoural tissues or in a particular areas of the tumour (Jensen and Whitehead, 2004). Finally, the RBP HuR, upon binding to RNA HuR, has been shown to stabilise it and alter its translation (Brennan and Steitz, 2001; Gorospe, 2003; Mazan-Mamczarz *et al*, 2003).

Another aspect is the differential expression of Tp73 transcripts in the peripheral and central areas of low-grade tumours as compared to high-grade gliomas that overexpress the gene in both areas. Beside the possibility of discriminating between high- and low-grade tumours, this result suggests that malignant cells from the periphery of low-grade gliomas differ from those located in the tumour centre. By using the two dimensional profiling strategy Li *et al* (2006) found a large number of genes that showed coordinated changes in transcript abundance and splicing, indicating that many distinct steps in gene expression from transcription, stability control and splicing may be coupled in different cell types (Li *et al*, 2006). In view of this observation it is tempting to speculate that the peripheral cells in low-grade tumours that differ from their central area congeners may evolve toward a more malignant stage or disappear to be replaced by tumour cells coming from the centre. We are considering the possibility of addressing these hypothesis by carrying out transcriptome analysis of tumour areas isolated by microdissection.

### Tp73 isoform prognostic value

A survival analysis aimed at determining the clinical relevance of the increase in ΔTAp73 isoform transcript levels in low-grade adult gliomas showed that patients with higher ΔEx2–3p73 contents in their tumours presented shorter survival than those with lower amounts of this transcript. Strikingly, increase in the TAp73 isoform transcript levels was also found in all patients, thereby arguing in favour of a dominant negative role for the ΔEx2–3p73 isoform in survival. This situation mimics that of ovarian tumours where a better overall survival was noted for patients exhibiting low expression of the ΔN’p73 isoform than for those with high expression, whatever the levels of TAp73 in the tumours (Concin *et al*, 2004). Moreover, ΔEx2–3p73 is a prognostic factor in low-grade gliomas even when age is considered.

Up until now, the status of the Tp73 gene involving its various isoforms has been examined in ovarian tumours, hepatocarcinomas, vulval cancers, oesophagus and gliomas (this report) (Ng *et al*, 2000; Cui *et al*, 2005). Although these studies point to increase in transcript levels of transdominant ΔTAp73 isoforms, it may be noted that, depending on the tumour pathology, a selective expression of one of the transdominant isoforms appears to be detected in certain tumour types, supporting the notion that among the various P1-generated isoforms, differential regulation exists. Given the necessarily limited number of patients included in this study, our data strongly suggest that the ΔEx2–3p73 isoform is selectively expressed in gliomas. It will be of interest to extend these observations to other tumour types in order to determine whether a more specific expression spectrum exists. Obviously, such selective patterns should reflect an abnormal distribution in tumour cells of the factors that contribute to splicing. In any case, whatever the identity of the expressed transdominant ΔTAp73 isoform, the biological significance directly relies on the neutralising role of this transdominant form to TAp73 and/or p53.

Although the gross natural history of adult low-grade glioma is known, a given individual outcome remains unpredictable. This lack of individual predictive factors results in daunting difficulties once therapeutic decisions are involved. When a low-grade glioma is newly diagnosed, clinicians have to decide when to proceed to treatment: not too early because therapeutic means – extensive surgical removal, radiation therapy, chemotherapy – are contestable as pertains to a benign tumour, but not too late either, that is before progression to higher grades of these infiltrating tumours. Up until now the indicators of such a change are clinical symptoms and imaging changes (e.g. contrast enhancement) but they only accompany, and fail to anticipate clinical tumour progression. New criteria allowing for earlier detection of tumour grade progression and correspondingly more expeditious therapeutic adaptation would be of heightened value as regards optimal treatment of these patients. Nevertheless, the use of ΔEx2–3p73 as such an indicator has one limitation, which is inherent to our methodology: as there exists no absolute quantification of this increase in transcript levels, it has got to be compared with a non-tumoural control group.

In conclusion, to the best of our knowledge this is the first report of a single molecular prognostic marker criterion for low-grade glioma survival in adults. This could lead to early treatment of the newly diagnosed adult patients harbouring low-grade gliomas and exhibiting increase in transcript levels of ΔEx 2–3p73.

## Figures and Tables

**Table 1:** (A) Tp73 global analysis aimed to show Tp73 involvement in this first series of patients. (B) Tp73 isoforms discrimination aimed to show specific isoform involvement on this second series of low-grade tumours patients only. (C) Normalised expression levels of Tp73 isoforms in non-tumoural (epileptic) and in low- and high-grade gliomas

	***n***	**Pathology**	**Biopsy-surgery**	**Age**
(A) – First series				
(*Tp73 involvement*)				
Low grades	11	AII: 6	9–2	43.5 (28–68)
		OD II: 5		
High grades	28	OD III: 7	6–22	60 (33–79)
		GBM: 21		
				
(B) – Second series				
(Tp73 isoforms)				
Low grades	23 (=11+12)	A II: 8	16–7	42.5 (28–68)
		OA II: 6		
		OD II: 9		
				

(C)				
	**Non tumoural tissue (normal expression)** *	**Low-grade gliomas medians (min-max)**		**High-grade gliomas medians (min-max) *n*=28**
		***n*=11**	***n*=23**	
P1-p73 (−Δ*C*_T_)	14.81	10.28 (7.33–16.83)		10.28 (4.86–15.72)
P1-p73 2^−ΔΔ*C*t^	1	23.05 (0.25–178.12)		23.09 (0.53–986.86)
ΔNp73 (−Δ*C*_T_)	13.84	14.36 (11.23–17.73)		13.52 (7.91–21.34)
ΔNp73 2^−ΔΔ*C*t^	1	0.69 (0.07–6.08)		1.24 (0.01–60.76)
TAp73 (−Δ*C*_T_)	14.16		8.21 (1.7–16.8)	ND
TAp73 2^−ΔΔ*C*t^	1		61.82 (0.15–5480.15)	
ΔEx2p73 (−Δ*C*_T_)	13.29		11.24 (5.31–17.14)	ND
ΔEx2p73 2^−ΔΔ*C*t^	1		4.14 (0.07 –252.48)	
ΔEx2–3p73 (−Δ*C*_T_)	11.94		7.48 (2.09–15.56)	ND
ΔEx2–3p73 2^−ΔΔ*C*t^	1		22.01 (0.08–922.88)	
ΔN’p73 (−Δ*C*_T_)	11.73		11.55 (6.62–16.43)	ND
ΔN’p73 2^−ΔΔ*C*t^	1		1.13 (0.04 –34.54)	

A=fibrillary astrocytoma; OA=oligoastrocytoma; OD=oligodendroglioma; GBM=glioblastoma.

II, III: tumour grades according to the World Health Organization.

Results are expressed in Δ*C*_t_ which is the threshold cycle differences (CtGAPDH-CtGene) and in 2^−ΔΔ*C*t^ which is the comparative threshold between non-tumoural and tumoural tissue where ΔΔ*C*_t_=Δ*C*_tTumoural_−Δ*C*_tNonTumoural_ and represents fold change value between tumoural and non-tumoural tissue. P1–p73, TAp73, ΔEx2p73 and ΔEx2–3p73 show significantly increased transcript levels as compared to non-tumoural epileptic tissues (*P*<10–3, *P*<10–4, *P*=0.01 and *P*=0.03, respectively). There is no difference of expression between low- and high-grade gliomas.

(^*^) Non-tumoural tissue value was based on the analysis of three epileptic patients. The mean of Δ*C*_t_ values was considered as normal expression. (ND: not done).

**Table 2:** Normalised expression levels of P1–p73 and ΔNp73 on the periphery of the tumour and at the centre of the tumour in low- and high-grade gliomas

	**High-grade gliomas median (min-max)**		**Low-grade gliomas median (min-max)**	
	**P1–p73**	**ΔNp73**	**P1–p73**	**ΔNp73**
Periphery of the tumour (−Δ*C*_T_)	9.67 (6.40–15.72)	13.20 (8.50–21.34)	12.16 (8.03–16.83)	14.83 (11.23–17.08)
Fold change between tumoural and non-tumoural tissue	44.92 (0.53–339.37)	1.55 (0.01–40.36)	7.33 (0.24–109.65)	0.69 (0.11 6.08)
Centre of the tumour (−Δ*C*_T_)	10.54 (4.86–15.50)	14.14 (7.91–21.12)	10.23 (7.33 –14.40)	14.36 (12.30–17.73)
Fold change between tumoural and non-tumoural tissue	19.44 (0.62–986.86)	0.81 (0.01–60.76)	23.87 (1.33–178.12)	0.69 (0.07–2.90)

Results are expressed in Δ*C*_t_ which is the threshold cycle differences (CtGAPDH-CtGene) and in 2^−ΔΔ*C*t^ which is the comparative threshold between non-tumoural and tumoural tissue where ΔΔ*C*_t_ =Δ*C*_tTumoural_−Δ*C*_tNonTumoural_ and represents fold change value between tumoural and non-tumoural tissue. There is a statistically significant difference for P1–p73 expression between the periphery of the tumour and the centre of the tumour for low-grade gliomas (*P*=0.031 in Wilcoxon test).
